# Effects of Cold Application on Chest Tube Removal Pain in Heart Surgery Patients

**Published:** 2018-01

**Authors:** Nooredin Mohammadi, Ali Pooria, Sajad Yarahmadi, Mohammad Javad Tarrahi, Hassan Najafizadeh, Payam Abbasi, Behzad Moradi

**Affiliations:** 1 Department of Critical Care Nursing, Nursing and Midwifery Faculty, Centre for Nursing Care Research, Iran University of Medical Sciences, Tehran, Iran,; 2 Department of Cardiac Surgery, Lorestan University of Medical Sciences, Khorramabad, Iran,; 3 Social Determinants of Health Research Center, Lorestan University of Medical Sciences, Khorramabad, Iran,; 4 Department of Bio-statistics and Epidemiology, School of Health, Isfahan University of Medical Sciences, Isfahan, Iran,; 5 Chronic Respiratory Diseases Research Center, National Research Institute of Tuberculosis and Lung Diseases (NRITLD), Shahid Beheshti University of Medical Sciences, Tehran, Iran.

**Keywords:** Chest tube, Cryotherapy, Pain, Visual analog scale

## Abstract

**Background:**

Chest tube removal is considered a painful technique, which may not respond well to palliative therapies. There are no standard procedures or guidelines to manage the pain associated with chest tube removal. This study aimed to examine the effects of cold application on pain reduction during and after chest tube removal.

**Materials and Methods:**

This randomized controlled trial was conducted on 90 hospitalized patients, undergoing heart bypass surgery at the intensive care units where at least a pleural chest tube was inserted. The patients were randomly divided into two groups (45 samples per group). In the cold application group, an ice bag was placed at the designated point for 20 minutes before chest tube removal, while only routine interventions were applied for chest tube removal in the control group. Pain severity was measured in the groups before, during, and 15 minutes after chest tube removal, using the visual analogue scale. Repeated measures ANOVA test was applied for data analysis.

**Results:**

There was no significant difference in the baseline pain score between the groups (P= 0.18). However, there was a significant difference in terms of pain severity score between the cold application (3.58±1.09) and control (4.73±0.86) groups during chest tube removal (P< 0.001). On the other hand, there was no significant difference between the groups regarding the score of pain severity at 15 minutes after chest tube removal (P= 0.38).

**Conclusion:**

Cold application, as a nonpharmacological intervention, may contribute to the alleviation of cryotherapy-related pain.

## INTRODUCTION

Cardiovascular diseases (CVDs) are among the most debilitating diseases, accounting for the highest rate of mortality worldwide. According to statistics, CVDs lead to death in more than 16 million people annually (1, 2). Cardiac surgery is the most frequent therapeutic intervention for ischemic and cardiac valve diseases (3). According to Texas Heart Institute, thousands of patients undergo cardiac and vascular surgeries in the United States every day (4). Also, 25 thousand heart surgeries are conducted in Iran per annum (5).

It is necessary to insert a chest tube in all types of heart surgeries in order to maintain the function of the heart and lungs and prevent pneumothorax, hemothorax, and pleural effusion. A chest tube is generally removed within 24 to 48 hours after cardiac surgery when the volume of secretion is less than 100–150 cc, and respiratory sounds are normal (6). Removal of a chest tube is a painful and frustrating experience for patients. Considering the chest tube tenacity to the surrounding tissues, its separation from the adjoined tissues is painful (7).

Medium to severe pain has been reported by patients during cryotherapy (CTR), while there are no standard procedures or guidelines to manage CTR-related pain (6, 7). Nurses do not generally employ any interventions to reduce CTR pain and do not realize when physicians remove the chest tube (8). Alleviation of pain caused by painful interventions, such as CTR, is usually based on the administration of narcotic drugs and nonsteroidal antiinflammatory drugs (NSAIDs). Although these drugs are highly efficient in pain alleviation, studies have shown that CTR is still a painful procedure for patients (9). Likewise, narcotic drugs and NSAIDs have some side effects, such as respiratory distress, nausea, itching, and gastrointestinal bleeding (10).

Today, many efforts are being made to reduce pain severity and dosage of narcotic medications, using nonpharmacological methods (11). Pain-relief methods, such as music therapy, touch therapy, diathermy, cold therapy, and acupuncture, are some of the nonpharmacological approaches to pain relief. These methods promote the patient’s autonomy and can be implemented using simple tools. Patients also show easy acceptance and good cooperation during treatment. Generally, these methods are not accompanied by the negative consequences or adverse effects of medicinal interventions (12).

Cold application is an efficient technique for pain relief. This technique reduces the speed of nervous conductance and pain. Similarly, based on the gate control theory of pain, stimulation of thick fibers through methods such as cooling may close the gate and reduce pain (13). Cold application can be used to reduce or reverse pain impulses via activating descending inhibitory neurons, which block ascending nociceptive nerves originating from the substantial gelatinosa (14).

Previous studies have investigated the effect of cold application on reducing pain owing to CTR. While cold application was reported to be effective in a study by Hassanzadeh et al. (14), Sauls reported its inefficacy (15). Obviously, the effect of cold application on reducing CTR-associated pain is not clear.

Accordingly, this study aimed to clarify the effects of cold application on relieving pain owing to CTR. In this study, effects of cold application on pain relief were examined during and after CTR in patients with at least a pleural chest tube. The study hypothesis was that pain severity scores would be lower in the cold application group, compared to the controls.

## MATERIALS AND METHODS

This randomized controlled trial was conducted from February 2016 to August 2016. The study was carried out in a single center, affiliated to the Cardiac Surgery Intensive Care Unit (CSICU) of Shahid Madani Hospital, Khorramabad, Iran. It is a specialized referral hospital for various types of cardiac surgeries, equipped with 100 beds and different departments, including echocardiography, angiography, angioplasty, electrophysiology, coronary care unit, and CSICU for men and women.

Ethical approval was obtained from the Ethics Committee of Iran University of Medical Sciences, Tehran, Iran. The study was also registered in the Iranian Registry of Clinical Trials (IRCT) under the code, IRCT2016012626217N1. The study sample comprised of all patients, who had undergone heart bypass surgery at Shahid Madani Hospital. Before enrollment in the study, an informed consent was obtained from each participant.

The inclusion criteria were as follows: 1) age range of 18–65 years; 2) having at least one pleural chest tube (not less than 24 hours or more than 120 hours should pass since insertion); 3) absence of vision problems or inability to give proper responses to the researcher’s questions; 4) no history of mental disorders; 5) lack of drug abuse or alcohol addiction; 6) body mass index (BMI) < 30 kg/m^2^; 7) no use of narcotic or sedative medications one hour before CTR; and 8) lack of supportive mechanical ventilation.

On the other hand, the exclusion criteria were as follows: 1) pain score above seven; 2) unwillingness to continue cooperation; 3) dissatisfaction during cold application; 4) use of narcotic or sedative medications during the intervention; and 5) sudden changes in the vital signs. The sample size was calculated based on a previous study by Mazloum et al. (13), which investigated the effects of ice bag application on pain intensity associated with chest tube removal following cardiac surgery. After the intervention, the mean (±standard deviation) pain intensity was 0.6±0.7, 2.0±2.6, and 1.9±1.9 in the ice bag, placebo, and control groups, respectively.

**Figure 1.**
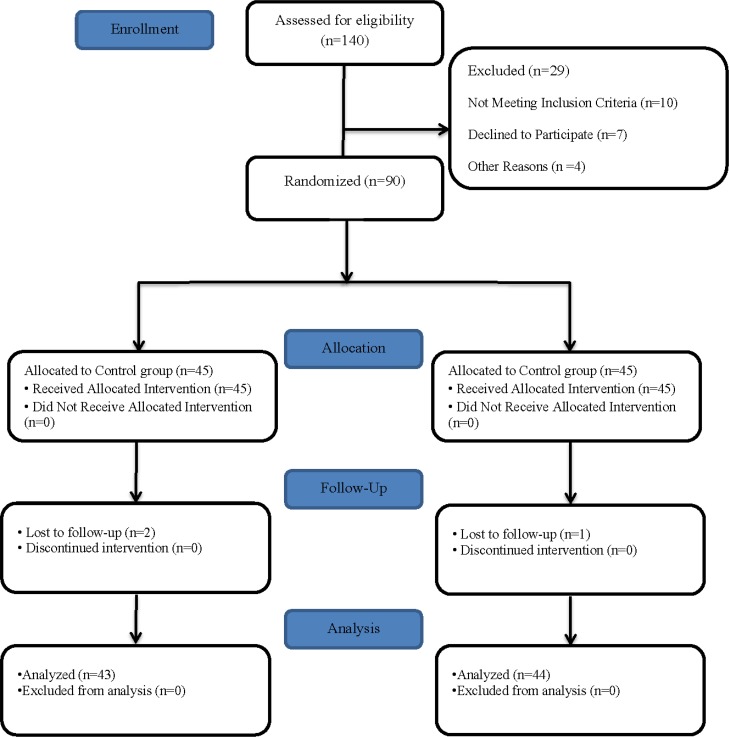
Consort Flow Diagram

Based on the mentioned study (β, 0.2; α, 0.05; S1, 2.3; S2, 2.7; μ1, 2.45; μ2, 4), the number of samples was estimated at 41 per group. However, we recruited 45 patients in each group to compensate for probable attrition and obtain more reliable results. The final sample size included 90 patients, who were admitted to CSICU for post-heart bypass surgeries. Ninety eligible patients were randomly allocated to either cold application or control group, using randomized fixed quadripartite blocks. Convenience sampling method was applied to assign samples to the groups; the participants had an equal chance of assignment to each of the groups. Figure 1 presents the study flow diagram.

A data recording checklist, consisting of two parts, was used for data collection. The first part was used to record demographic data (10 questions). Also, a checklist was developed to review relevant literature; its content validity was confirmed by 10 faculty members from Iran University of Medical Sciences. The second part of the checklist evaluated pain severity, using a visual analogue scale (VAS). In this tool, a score of zero indicates no pain, while a score of 10 presents the worst pain imaginable. Accordingly, the participants indicated the severity of pain on VAS. Overall, numerous studies have used this scale to measure pain severity in heart surgery patients (7, 14–16). In the present study, the participants were familiarized with VAS instructions and methods before CTR.

Surgery was performed by one surgeon, and surgical methods were similar for all patients. Anesthesia was subsequently induced in both groups, based on the standard protocol for heart surgery. The diameter of chest tubes was also similar in both groups (F= 14–16). Before the intervention, the participants were given some information about cold application. The chest tubes were removed using a similar technique under the clinical supervision of five experienced CSICU nurses. Data were collected by another nurse, who was not a member of the research team to prevent any possible bias in data collection.

The patients were placed in the semifowler position with a pillow under their head and shoulder to achieve more comfort during the intervention; their rough cloths were also removed. In the cold application group, a 9-inch ice bag (Easy Life Inc., China) was placed over the chest tube at −5°C; it remained in contact with the patient’s skin for 20 minutes. A layer of sterile gauze was then placed over the chest tube and skin to prevent direct contact with the ice bag. The chest tube was removed maximally two minutes after lifting the ice bag. On the other hand, the control group did not receive cold therapy, and routine interventions were applied according to the ward policies. The pain score was measured by VAS in the two groups before, during, and 15 minutes after CTR.

The collected data were analyzed using descriptive tests in SPSS version 21. Descriptive statistics were used to summarize the demographic information. Chi square and *t* test were also used to compare demographic data. Repeated measures ANOVA was applied to compare pain scores between the groups at different intervals. In addition, ANOVA test was applied for comparing pain scores between the groups at any time interval. *P*-value less than 0.05 was considered statistically significant.

## RESULTS

In the present study, 90 eligible subjects were divided into the cold application and control groups. The cold application group received cold therapy before CTR, while the control group received routine interventions according to the ward policies. In a total of 90 participants, two patients from the cold application group and one patient from the control group were excluded for having a pain score above seven. After excluding these cases from the survey, data from 87 patients were finally analyzed.

The mean age of the participants was 57.87±8.8 years in this study, and 65.5% of the subjects were male. Most of the participants underwent coronary artery bypass grafting (CABG) (79.3%). There was no significant difference between the groups with regard to the baseline characteristics, including gender, BMI, education, occupation, marital status, type of surgery, history of chronic pain before surgery, history of regular sedative drug use before surgery, or history of chest tube insertion before surgery (*P*> 0.05) (Table 1).

**Table 1.** Comparison of baseline and demographic characteristics among the study groups^a^

**Characteristics**	**Cold**	**Control**	**P value**
**Age, y**	58.9±8.3	56.8±9.2	P=0.26^b^ t=1.11
**Gender**			P=0.93^c^
Female	15(34.9%)	15(34.1%)	
Male	28(65.1%)	29(65.9%)	
**BMI**			P=0.88^b^
18.5>	2(4.7%)	3(6.8%)	
18.8–24.9	22(51.2%)	21(47.7%)	
25–29.9	19(44.2%)	20(45.5%)	
**Education**			P=0.32^c^
Illiterate	9(20.9%)	16(36.4%)	
Under Diploma	18(41.9%)	17(38.6%)	
Diploma	11(25.6%)	9(20.5%)	
University	5(11.6%)	2(4.5%)	
**Employment status**			P=0.92^c^
Unemployed	3(7%)	3(6.8%)	
Housewife	8(18.6%)	10(22.7%)	
Working	22(51.2%)	23(52.3%)	
Retired	10(23.3)	8(18.2%)	
**Martial Status**			P=0.37^c^
Single	1(2.3%)	4(9.1%)	
Married	34(79.1%)	33(75%)	
Divorced	0(0%)	1(2.3%)	
Wife feet	8(18.6%)	6 (13.6%)	
**Type of surgery**			P=0.26^c^
Valve surgery	11(25.6%)	7(15.9%)	
CABG	32(74.4%)	37(84.1%)	
**History of chronic pain**			P=0.42^c^
YES	6(14%)	9(20.5%)	
NO	37(86%)	35(79.5%)	
**History of taking analgesics**			P=0.47^c^
YES	6(14%)	4(9.1%)	
NO	37(86%)	40(90.9%)	
**Experienced chest tube**			P=0.22^c^
YES	5(11.6%)	2(4.5%)	
NO	38(88.4%)	42(95.5%)	

Abbreviations: BMI, Body mass index

a Data are presented as No. (%) or mean ± SD.

b Independent t-test.

c Chi-square test.

**Figure 2.**
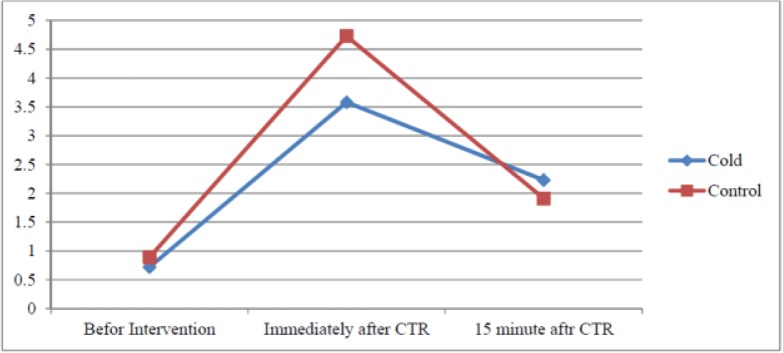
Trend of changes in pain intensity in the study groups during the study

Based on the findings, gender, age, BMI, history of chronic pain, history of analgesic use, and chest tube insertion had no effects on pain due to CTR in the cold application and control groups (*P*> 0.05). The mean pain severity scores of the groups are presented in Table 2 and Figure 2, based on the time of CTR-related pain measurement. The difference (0.32) in the baseline pain severity scores was not significant between the groups (*P*= 0.18). In addition, the mean pain severity scores were compared between the groups immediately after CTR, and the difference (1.15) was found to be significant (*P*< 0.001). However, the difference (0.17) in the mean pain severity score was not significant between the groups at 15 minutes after CTR (*P*= 0.38).

**Table 2.** Comparison of pain intensity scores among the study groups at different time^a^

**Study Group**	**Before intervention Mean±SD**	**Immediately after CTR Mean±SD**	**15min after CTR Mean±SD**	**Repeated measures analysis Mean±SD**
**Cold**	2.23±1.02	3.58±1.09	0.72±0.79	P<0.001
**Control**	1.91±1.21	4.73±1.08	0.89±0.97	P<0.001
**P-value**	P=0.18	P<0.001	P=0.38	P<0.001

a Data are presented as mean ± SD.

## DISCUSSION

Chest tube removal is considered a painful and debilitating experience for all patients (7). However, there are no standard procedures or guidelines to manage CTR-related pain (17). It is proposed that treatment with nonpharmacological methods, such as cold therapy, can alleviate the pain caused by irritating procedures. Cold application decelerates tissue metabolism and nerve transfer speed locally and exhibits vasoconstrictive, antiinflammatory, antispasmodic, and analgesic effects (14).

Despite the promising results of different studies on the effects of cold application on reducing pain caused by CTR, other clinical trials have reported conflicting results (8, 14, 15). The findings of the present study showed that cold application might be effective in reducing pain during CTR. Pain intensity was compared between the groups immediately after CTR, before CTR, and 15 minutes after CTR. The results confirmed that CTR is still a frustrating procedure for patients. Different studies have reported similar results in this area (8, 13, 14, 18).

In line with the present study, several investigations have shown that cold application is effective in relieving pain during CTR (7, 14, 18, 19). In our study, pain severity during CTR was significantly lower in the cold application group, compared to the controls. This finding is consistent with the results of a study by Demir and Khorshid (7), in which 90 patients with chest tubes were randomized and assigned to cold application (20 minutes), placebo, and control groups; pain severity was measured by VAS. All patients received pain medications before CTR. The pain scores significantly decreased in the intervention group. One can assume that use of an ice bag for 20 minutes accounts for the consistent results, as the bag needs to be applied for at least 20 minutes to induce favorable cryogenic effects (20).

On the other hand, Sauls reported cold application to be ineffective in reducing pain caused by CTR (15). This discrepancy can be explained by differences in the parameters evaluated in the current study. Also, the shorter duration of ice bag application (10 minutes) and larger sample size, as opposed to the study by Sauls, might be influential. In another study by Hasanzadeh et al., implementation of three interventions (inhalation of lavender essential oil, cold application, and combination of cold application and lavender essential oil inhalation) was identically effective in relieving pain and anxiety due to CTR. In their study, cold application was discarded when chest tube temperature reached 13°C, while in this study, cold was applied at the point of chest tube for 20 minutes (14).

The results of the present study showed that cold application has no effects on reducing pain severity at 15 minutes after CTR. This finding is not consistent with the results of previous research (9, 13, 14, 16, 18) and shows that implementation of nonpharmacological techniques for pain relief might be efficient in reducing pain severity at 15 minutes after CTR. In the previous studies (9, 14, 16, 18), oral or injectable sedative treatment was applied, along with nonpharmacological interventions, while in the current study, patients received no sedative treatments, based on the instructions on the intervention day. With respect to the long half-life of drugs versus the effective period of nonpharmacological therapies, it can be deduced that pain scores reduced at 15 minutes after CTR because of medicinal therapies.

In a study by Mazloum et al., no sedative treatment was applied for patients on the day of intervention. Unlike our study, the results indicated that cold therapy was effective in pain relief at 15 minutes after CTR (13). In addition, pain assessment in patients with mediastinal chest tubes rather than pleural tubes in the mentioned study can account for this contradiction, as pleural tubes produce more pain than mediastinal tubes given the presence of many sensory nerves on the surface of pleural membranes. Also, pain varies in different anatomical regions. In the study by Mazloum et al., two chest tubes were conseucutively removed in less than 30 minutes; therefore, the execution method in the present study was different from the mentioned research (13).

The present study was carried out with an open-label design, as the nature of intervention made it impossible to integrate a proper placebo. The limitations of this study include execution of CTR by five different individuals. Of course, we tried to reduce the effect of this factor by providing training on a single CTR technique. Finally, the patients might express different responses to pain with respect to the subjective nature of pain and their physical, emotional, and cultural status. This limitation can create some bias in the results, although such potential biases were minimized by means of randomization.

It is hoped that the present findings promote future studies in this area. It is recommended to conduct a study with a larger sample size on CTR-associated pain in non-heart bypass surgery patients in order to confirm the present findings.

## CONCLUSION

The results of this randomized controlled trial suggest that cold application, as a nonpharmacological intervention, may temporarily contribute to the alleviation of pain caused by CTR.
